# MDSC depletion during immunization with heat-killed *Mycobacterium tuberculosis* increases protection against BCG infection

**DOI:** 10.3389/fimmu.2025.1646526

**Published:** 2025-07-31

**Authors:** Arpa Aintablian, Anna M. Arold, Haisam Alattar, Laura Cyran, Christoph Schoen, Nelita Du Plessis, Gerhard Walzl, Ulrich Schaible, Andreas Beilhack, Natalie E. Nieuwenhuizen, Manfred B. Lutz

**Affiliations:** ^1^ Institute for Virology and Immunobiology, University of Würzburg, Würzburg, Germany; ^2^ Department of Medicine II, Würzburg University Hospital, Würzburg, Germany; ^3^ Department of Microbiology and Immunology, Faculty of Pharmacy, Assiut University, Assiut, Egypt; ^4^ Institute for Hygiene and Microbiology, University of Würzburg, Würzburg, Germany; ^5^ Department of Science and Technology/National Research Foundation (DSI-NRF) Centre of Excellence for Biomedical Tuberculosis Research, South African Medical Research Council Centre for Tuberculosis Research, Biomedical Research Institute, Division of Molecular Biology and Human Genetics, Faculty of Medicine and Health Sciences, Stellenbosch University, Cape Town, South Africa; ^6^ Division Cellular Microbiology, Research Center Borstel, Borstel, Germany; ^7^ German Center for Infection Research (DZIF), Site Hamburg-Luebeck-Borstel-Riems, Borstel, Germany

**Keywords:** MDSC, *Mycobacterium tuberculosis*, BCG, vaccines, ATRA

## Abstract

Tuberculosis (TB) remains one of the deadliest infectious diseases globally. Although the approved human Bacille-Calmette-Guérin (BCG) vaccines provide limited protection, a vaccine based on *Mycobacterium tuberculosis* (Mtb) has yet to be approved. Our previous findings demonstrated that s.c. immunization with heat-killed Mtb significantly increased the number of monocytic myeloid-derived suppressor cells (M-MDSC) in mice. Thus, we hypothesized that the defense against a subsequent BCG infection would be compromised in Mtb-immunized mice. Surprisingly, mice vaccinated with Mtb were protected against BCG infection and exhibited elevated frequencies and activation of dendritic cells (DC) and mycobacteria-specific T cells, despite high frequencies and suppressor activity of M-MDSC. Genetic ablation of CCR2^+^ monocytic cells or pharmacological intervention with all-trans retinoic acid (ATRA) reduced the frequency of Mtb-induced M-MDSC, enhanced the frequencies and activation of DC and CD4^+^ T cells, and resulted in decreased bacterial loads in the lungs and spleen. These findings provide new insights into TB vaccination using heat-killed Mtb despite the concurrent unwanted effects of vaccine-induced M-MDSC. M-MDSC depletion via ATRA further shifts the balance toward immunity and should be considered an adjunct host-directed therapy alongside TB vaccines in humans.

## Introduction

Tuberulosis (TB), an infectious disease caused by *Mycobacterium tuberculosis* (Mtb), still accounts for a significant burden of annual morbidity and mortality worldwide. Mtb-based vaccine candidates have been shown to induce T cell responses but have so far failed to lead to an approved vaccine (1). Currently, multiple Mtb-based vaccine candidates are in various phases of clinical trials (2). Bacille-Calmette-Guérin (BCG), the live attenuated form of *Mycobacterium bovis* (Mbov), which causes TB in cattle, is the only available FDA-approved TB vaccine to date. In many countries, BCG is routinely administered to infants shortly after birth, effectively preventing meningeal and miliary TB and displaying 60-80% protective efficacy against pulmonary disease in children (3). However, the BCG vaccine shows limited and highly variable protective efficacy in adults, representing the most active TB cases (2, 4). While vaccines are typically generated from the same microbe, the BCG vaccine represents a heterologous vaccine, lacking numerous antigenic determinants compared to Mtb (5). However, since Mtb has developed many immune evasion mechanisms preventing immune responses, especially immune memory, BCG currently appears to represent the best compromise for a TB vaccine (6). The challenge remaining is to apply Mtb as a vaccine but circumvent critical immune evasion mechanisms preventing the immune memory and, thereby, the vaccine success.

As a novel immune evasion mechanism of Mtb, we found an accumulation of myeloid-derived suppressor cells (MDSC) in the blood of TB patients and healthy individuals recently exposed to Mtb (7). Similarly, suppressive MDSC were detected in the lungs of Mtb-infected mice, providing a suitable environment for pathogen survival (8). Two major MDSC subsets have been classified as CD11b^+^ Ly6C^high^ Ly6G^-^ monocytic MDSC (M-MDSC) and CD11b^+^ Ly6C^low^ Ly6G^+^ granulocytic MDSC (G-MDSC, also known as polymorphonuclear (PMN)-MDSC), determined by their monocytic or granulocytic hematopoietic origin, respectively (9–12). MDSC possess different immunosuppressive mechanisms. These include T cell receptor (TCR) nitrosylation, attributed to their expression of intracellular inducible nitric oxide synthase (iNOS) and consequent release of nitric oxide (NO), and T cell metabolic starvation due to arginase 1 (Arg1)-dependent catabolism of arginine from the extracellular space (13, 14).

Our previous work showed that even a heat-killed Mtb prime-boost vaccine massively induced M-MDSC (15). These M-MDSC accumulated in the spleen and displayed potent dendritic cell (DC)-killing capacity in an iNOS-dependent manner *in vivo*, thereby indirectly suppressing effector T cell responses (15). Overall, it is remarkable that the heat-killed Mtb vaccine still shows the same immune evasion mechanism of M-MDSC induction as the live infection. Further, it remains unknown whether a heat-killed BCG or a heat-killed Mbov vaccine also induces the differentiation of monocytes into suppressive M-MDSC, resulting in insufficient protection. In fact, few reports exist about the protective innate and/or adaptive immune effects of BCG vaccination on a subsequent Mbov infection in mice (16). Despite differences among mycobacterial species, most immune effects against challenges have been described with heterologous Mtb for mice, while BCG vaccination has been tested frequently against Mbov infection in cows. However, in cattle, immune responses were scarcely followed due to the limited availability of reagents. Most experimental studies showed a reduced disease severity, but field trials also showed protection from infection (17, 18).

Host-directed therapies targeting MDSC against tumors show promising results in animal models and clinical trials with cancer patients (19, 20). Since chronic infectious diseases such as HIV or TB still suffer from the lack of effective vaccines, the extrapolation of host-directing therapies targeting MDSC are also discussed for these infections (21). All-trans retinoic acid (ATRA), an active metabolite of vitamin A, has been shown to interfere with MDSC development by shifting it towards pro-inflammatory myeloid effector cells (22). Promising outcomes have been observed in murine Mtb infection models (8, 23–25) and cancer patients (26, 27). Since ATRA was also successfully combined with cancer vaccines in patients (28) but has not yet been tested in combination with an Mtb vaccine, we applied it here in our mouse model.

To gain further insights into the conditions favoring vaccine-induced M-MDSC by mycobacteria, we altered multiple factors in an established immunization protocol (15), including the prime-boost immunization interval, type of adjuvant, vaccine dose, and method of Mtb preparation, as well as the use of different heat-killed bacterial species within the vaccine. We also investigated the effect of heat-killed Mtb and BCG vaccines on subsequent live BCG infection in mice. Our results indicate that Mtb appears quantitatively superior to other bacteria tested here in the induction of M-MDSC. Surprisingly, an intranasal challenge of Mtb-immunized mice with BCG resulted in simultaneous iNOS-dependent M-MDSC-mediated suppression and effector immune responses by DC and T cells in the lungs, biased towards protective effects. Genetic CCR2-targeted depletion or pharmacological interference with M-MDSC by ATRA reduced their frequencies, enhanced DC and CD4^+^ T cell frequencies, and further reduced bacterial burdens in the lungs and spleen. These results provide new insights into the relationship between M-MDSC-driven suppression and DC/T cell-mediated immunity. Combining Mtb vaccination with ATRA treatment may be a new strategy for developing an effective Mtb-based TB vaccine in humans.

## Results

### Mtb outcompetes *Mycobacterium smegmatis* and Listeria in M-MDSC induction, in contrast to BCG, which fails to induce M-MDSC

The Mtb strain H37Ra is the key component of Complete Freund's Adjuvant (CFA), a commercially available emulsion of heat-killed and lyophilized Mtb H37Ra in Incomplete Freund's Adjuvant (IFA). This formulation is widely used as a vaccine adjuvant in animal models (29, 30). While IFA has entered human clinical studies as a vaccine adjuvant (Montanide) (31–33), CFA is not utilized in humans due to its severe side effects, despite having stronger adjuvant properties (30). Our previous findings indicated that double injections of commercial CFA or in-house prepared heat-killed Mtb H37Ra in IFA resulted in potent systemic M-MDSC induction in mouse spleens (15), a consequence not desired in an adjuvant. Since IFA did not induce M-MDSC, it was determined that the mycobacterial component was responsible for this effect (15). The question remained whether M-MDSC induction is an inherent feature of Mtb, or if other mycobacteria would also exhibit this effect when applied under the same conditions of heat-killing and s.c. application.

**Figure 1:**
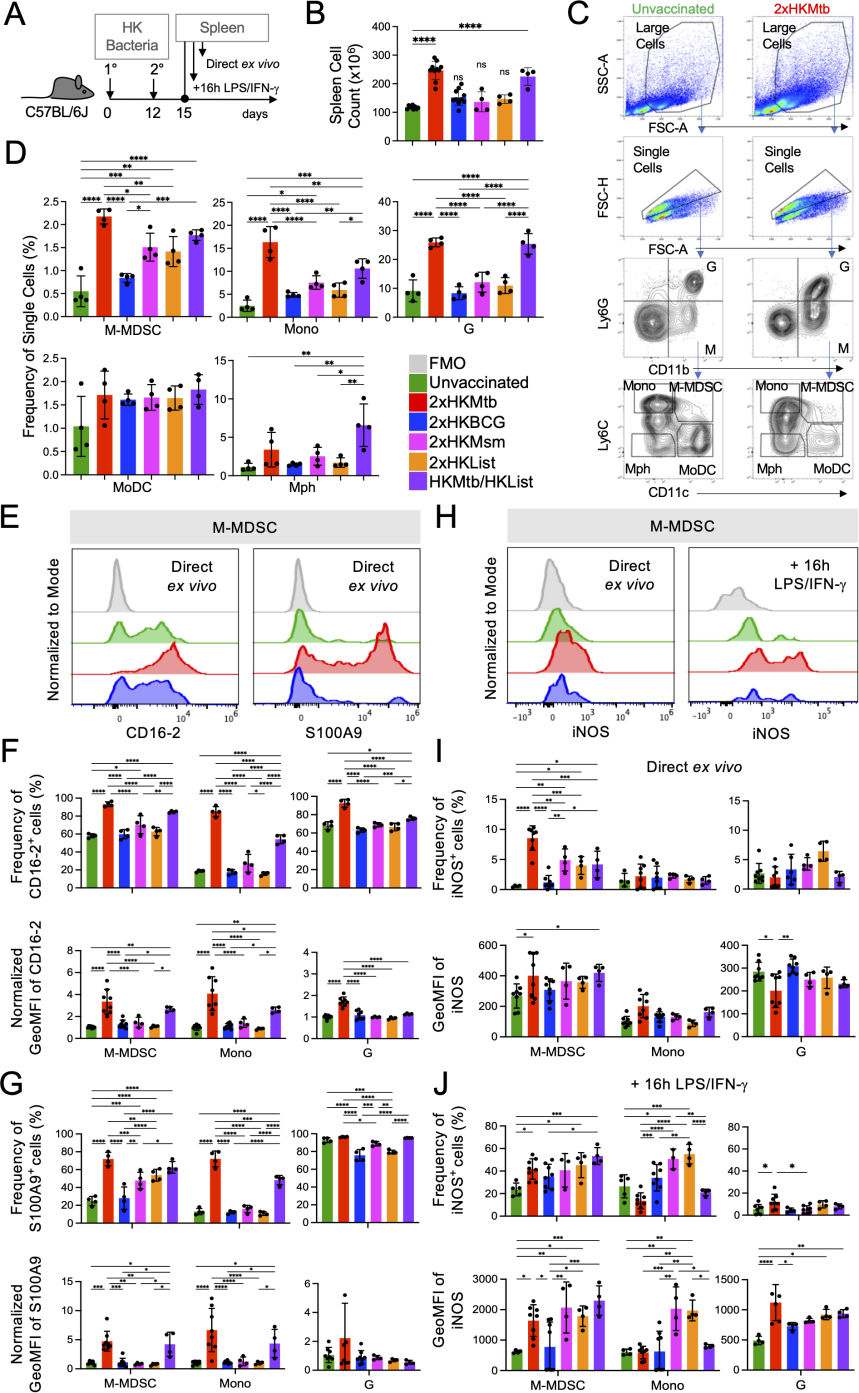
Superior potential of 2xHKMtb immunization over other bacteria to increase M-MDSC frequencies, activation and iNOS expression **(A)** Time scale of the standard protocol for primary and secondary immunization. Mice remained unvaccinated or were immunized s.c. twice with 10^6^ CFU of the indicated heat-killed (HK) bacteria in IFA at day 0, followed by a booster dose at day 12. Spleens were harvested and analyzed at day 15. **(B)** Spleen cell numbers obtained from the indicated immunization conditions. **(C)** Gating strategy to identify granulocytes **(G)**, M-MDSC, monocytes (Mono), macrophages (Mph), and monocyte-derived DC (MoDC). **(D)** Frequencies of splenic cell types indicated in **(C, E).** Representative histograms showing expression of CD16–2 and S100A9 activation markers in M-MDSC gate. **(F, G)** Frequency and normalized GeoMFI of CD16–2 and S100A9 in M-MDSC, Mono and G gates, respectively, directly *ex vivo* **(H)** Spleen cells were restimulated *in vitro* by LPS/IFN-g for 16h then stained for iNOS. Histograms showing iNOS expression directly *ex vivo* and after restimulation *in vitro* in M-MDSC gate. **(I, J)** Frequency and GeoMFI of iNOS in M-MDSC, Mono and G gates directly *ex vivo* **(I)** and after restimulation **(J, B, D)** Statistics by ordinary one-way ANOVA with multiple comparisons and Tukey's post test. n=4–10 biological replicates, 2–4 independent experiments. **(F, G, I, J)** Statistics by two-way ANOVA with multiple comparisons and Tukey's post test. n=3–8 biological replicates, 2–4 independent experiments. *p<0.05; **p<0.01; ***p<0.001; ****p<0.0001.

Here, we examined M-MDSC induction by double immunization with different heat-killed (2xHK) bacteria in IFA, including BCG, *Mycobacterium smegmatis* (Msm), Mbov, and as a different species of intracellular bacteria *Listeria monocytogenes* (List), and single vaccination with Mtb followed by List (**Figure 1A**). As observed before, splenomegaly occurred after 2xHKMtb immunization, which was also seen here after immunization with Mtb/List, but not with the other double vaccines (**Figure 1B**).

Spleens were further analyzed by flow cytometry for monocytic and granulocytic cells, which could be distinguished according to their relative surface expression of CD11b and Ly6G. Monocytic cells were further subdivided into Ly6C^high^ CD11c^-^ classical monocytes (Mono), Ly6C^high^ CD11c^+^ M-MDSC, Ly6C^-^ CD11c^+^ monocyte-derived DC (MoDC), and Ly6C^-^ CD11c^-^ macrophages (Mph) (**Figure 1C**), as described earlier. Frequencies of M-MDSC, Mono, and granulocytic cells (G) comprising of almost exclusively neutrophils, dramatically increased with the 2xHKMtb and Mtb/List vaccines, while only to a lower extent with 2xHKMsm and 2xHKList (**Figure 1D**). Interestingly, 2xHKBCG immunization did not significantly increase the spleen cellularity and frequencies of M-MDSC, classical monocytes, and granulocytes compared to untreated controls (**Figures 1B, D**). Mph and MoDC showed only marginal differences between the groups (**Figure 1D**) and were not followed further.

To further characterize the activation state of the myeloid cell subsets, we used the CD16–2 and S100A9 markers, which were found to be transcriptionally induced in M-MDSC by several authors before (34). CD16–2 is the activating FcγRIV (35), and S100A9 belongs to the family of alarmins (36), both constitutively expressed by neutrophils and G-MDSC but not monocytes. We demonstrate here that both markers correlate with the upregulation of iNOS in M-MDSC after Mtb contact, and therefore could be used as additional markers indicating the activation of M-MDSC. 2xHKMtb and Mtb/List immunizations strongly induced the expression of CD16–2 and S100A9 by M-MDSC, Mono, and G populations, compared to vaccines from all other bacterial species (**Figures 1E–G**). Conversion of monocytes into suppressive MDSC with NO-releasing capacity is a two-step process that first requires an initial 'licensing' and expansion step, e.g., by GM-CSF or bacterial immunization. Upon secondary challenge, e.g., by LPS/IFN-γ, functional suppression is activated by up-regulation of iNOS and release of NO. Therefore, we analyzed iNOS expression directly and after re-stimulation of splenocytes *in vitro* with LPS/IFN-γ. Immunization with 2xHKMtb resulted in a strong increase in the frequency of iNOS^+^ M-MDSC, whereas 2xHKMsm, 2xHKList, and Mtb/List only moderately, in contrast to 2xHKBCG which had no effect (**Figures 1H, I**). Re-stimulation with LPS/IFN-γ strongly increased iNOS expression levels and frequencies of iNOS^+^ M-MDSC in most immunization groups. Still, the expression levels of iNOS remained low in cells from BCG-immunized mice (**Figures 1H, J**).

Due to the unique ability of 2xHKMtb to induce M-MDSC, we tested how the bacterial dose, the immunization interval, or the adjuvant type influenced the myeloid cell compartment and M-MDSC induction. Immunization of mice with 100-fold lower Mtb doses reduced spleen cell numbers and frequencies of classical monocytes but did not affect the frequency of M-MDSC and granulocytic cells compared to our standard immunization protocol (**Supplementary Figures 1A, D**). Application of the booster vaccine after longer intervals (**Supplementary Figures 1B, E**) or the use of aluminium hydroxide (alum) instead of IFA (**Supplementary Figures 1C, F**) had no significant effect on spleen cell numbers or M-MDSC and granulocyte frequencies. Still, it resulted in lower numbers of monocytes compared to our standard immunization protocol. To investigate whether the method of Mtb preparation or the attenuation of BCG affects M-MDSC induction, we compared vaccines prepared from heat-killed and lyophilized Mtb to those prepared with heat-killed but non-lyophilized Mtb (MtbM). Also, comparisons were made between BCG and its non-attenuated counterpart, Mbov. Vaccination with 2xHKMtbM led to lower frequencies of monocytes and granulocytic cells than 2xHKMtb but similar or only slight reductions in spleen cell counts and M-MDSC frequencies and activation markers (**Supplementary Figures 2A-H**). 2xHKMbov immunization resulted in similar frequencies of M-MDSC to 2xHKBCG and was comparable in most readouts, apart from higher levels of CD16–2 on granulocytic cells and iNOS+ on directly analyzed M-MDSC (**Supplementary Figures 2A-H**). This indicates that neither the Mtb preparation nor the attenuation of Mbov to BCG has a major influence on the capacity of these strains to induce M-MDSC. Together, these results suggest that intrinsic components of the Mtb bacterial strain may be responsible for inducing M-MDSC.

### Mtb immunization before BCG infection induces lung M-MDSC but reduces lung bacterial load, in contrast to BCG vaccination

**Figure 2:**
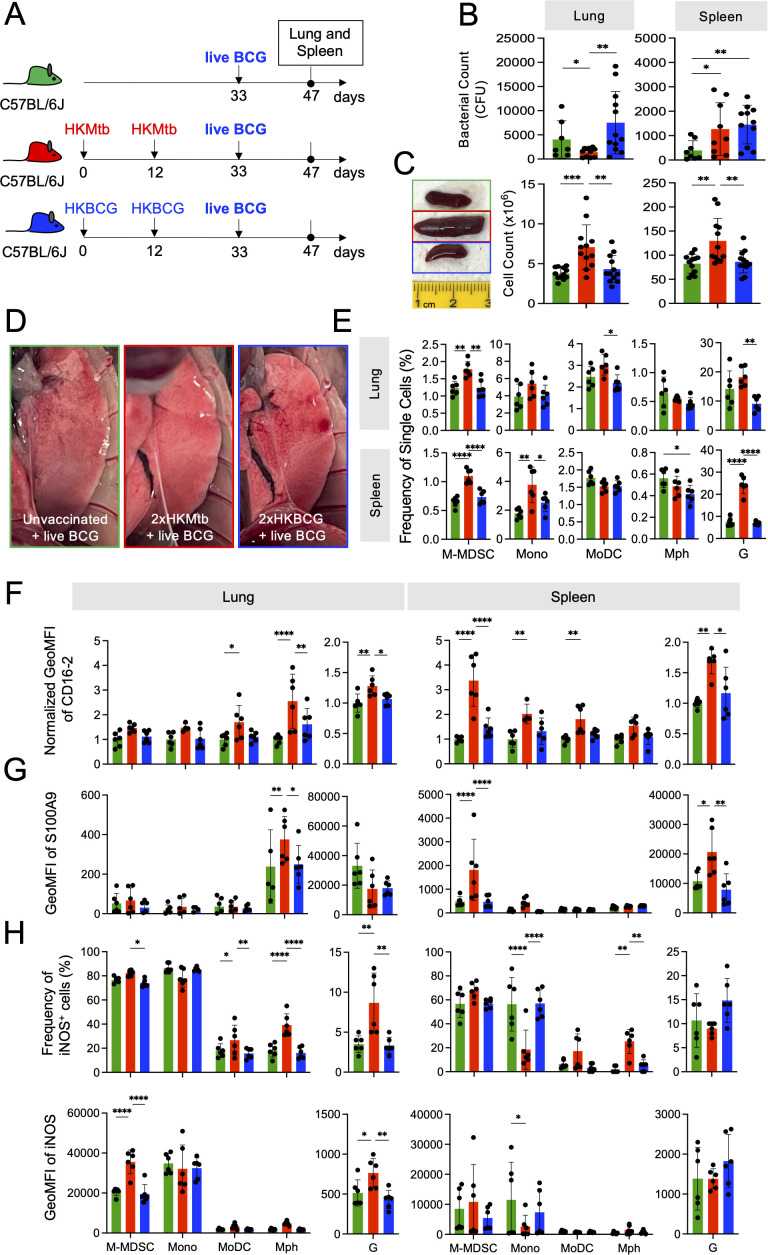
Live BCG-infected lungs of 2xHKMtb- but not 2xHKBCG-immunized mice show increased lung infiltration of M-MDSC, but they lose activation and suppression markers. **(A)** Scheme showing 2x immunization protocols with subsequent live BCG infection. Mice remained untreated or were immunized s.c. with 10^6^ CFU heat-killed (HK) bacteria in IFA at day 0 followed by a booster dose at day 12. On day 33, all mice were infected i.n. with 10^7^ CFU live BCG; lungs and spleens were harvested and analyzed at day 47. **(B)** Bacterial loads in whole lung and spleen. **(C)** Representative spleen photographs and cell numbers of lung and spleen as indicated. **(D)** Representative photographs of 12 lungs per group showing reduction in areas of inflammation (red/white patches) in 2xHKMtb-immunized mice. **(E)** Frequencies of myeloid cells in specified gates of lung and spleen. Gates as in **Figure 1C**. **(F, G)** Expression of CD16.2 and S100A9 by the indicated cell types via flow cytometry. **(H)** Lung and spleen cells were restimulated *in vitro* by LPS/IFN-g for 16h then stained for iNOS. **(B)** Statistics by unpaired, two-tailed t-test. n=8–10 biological replicates, 2 independent experiments. **(C, E-H)** Statistics by ordinary one-way ANOVA with multiple comparisons and Tukey's post test. Data from n=6 mice with 2 technical replicates from 2 independent experiments. *p<0.05; **p<0.01; ***p<0.001; ****p<0.0001.

Next, we sought to investigate the influence of 2xHKMtb or 2xHKBCG immunizations, which contrasted in their capacity to induce M-MDSC, on a subsequent BCG infection. Mice were immunized accordingly or remained untreated and were infected i.n. with live BCG three weeks after the booster vaccination (**Figure 2A**). There was a reduction in bacterial load in the lungs but an increase in the spleen after 2xHKMtb immunization compared to unvaccinated mice (**Figure 2B**). Surprisingly, 2xHKBCG vaccinated animals showed a similar increase in bacterial load in the spleen and a non-significant trend for higher bacterial load in the lungs (**Figure 2B**). As observed with 2xHKMtb immunization already before infection (15), visible splenomegaly persisted also after BCG infection in this group of mice, correlating with increased cell counts in the spleen and lungs (**Figure 2C**). The unexpected protective effect of 2xHKMtb immunization, despite M-MDSC induction, was confirmed by anatomical examination of the lungs, which appeared clearer with fewer patches of inflammation in 2xHKMtb vaccinated animals (**Figure 2D**). 2xHKMtb immunization before BCG infection enhanced the frequencies of M-MDSC, monocytic, and granulocytic cells in the lungs (**Figure 2E**). Extramedullary myelopoiesis induced by 2xHKMtb immunization persisted after BCG infection, shown by increased numbers of M-MDSC, monocytes, and granulocytic cells in the spleen (**Figure 2E**).

Flow cytometric analysis of the spleen after BCG infection revealed two cell populations with differential Ly6G and CD11b expression levels within the G gate, which we designated G^high^ and G^low^ (**Supplementary Figure 3A**). Cells in the G^high^ gate expressed higher levels of S100A9 and CD16–2 markers than those in the G^low^ gate, but only moderate differences appeared in the frequencies and expression levels of iNOS and Arg1 suppression markers (**Supplementary Figures 3B, C**). Overall, the cells in the G^high^ and G^low^ gates of the 2xHKMtb-immunized group appear to reflect differentiation or activation states, with the G^high^ cells being functionally more advanced than the G^low^ population. These data agree with our previous findings, where the G^high^ and G^low^ cells reflected mature and immature neutrophils in the spleen, respectively, as judged by their nuclear shape (37). Thus, the total G gate was used for further analyses. We also investigated the activation and functional state of alveolar macrophages (AM) in our model, as they are the first immune cells to encounter mycobacteria in the lungs and are critical in determining disease outcome (38). The CD11c^+^ fraction within the Ly6G^-^ CD11b^-^ (dn) gate was designated as AM (**Supplementary Figures 3D, E**). The CD11b^-^ Ly6G^-^ G^low^ population in the lungs expressed low levels of CD11c and showed a dispersed forward and side scatter profile. Hence, it did not appear to represent immature neutrophils as in the spleen (**Supplementary Figure 3F**). Thus, only the G^high^ population was analyzed in the lungs. Analyses of AM's frequency, activation, and suppression markers showed no differences between immunization groups (**Supplementary Figures 3G, H, I**). This agrees with recent findings that mycobacterial infection switches from AM to monocyte-derived Mph after a few days (39).

We then studied the activation state of the monocytic and granulocytic cells induced by live BCG infection directly *ex vivo*. While 2xHKMtb immunization followed by BCG infection resulted in up-regulation of the markers CD16–2 and S100A9 and down-regulation of iNOS in splenic M-MDSC and monocytes, this was not observed in the lungs (**Figures 2F–H**). Instead, Mph, MoDC, and granulocytes showed higher expression levels of these markers (**Figures 2F, G**). Although increased frequencies of iNOS^+^ Mph and MoDC were observed after *in vitro* restimulation, no improvement of iNOS expression per cell was found as indicated by GeoMFI, in contrast to M-MDSC and granulocytes (**Figure 2H**). The activation and expansion of iNOS^+^ Mph and granulocytes may indicate that they have achieved immunogenic properties, based on findings that intracellular killing of mycobacteria by macrophages is iNOS-dependent (40). This may point to a switch from a dominant suppressive myeloid compartment towards immunogenic but iNOS^+^ myeloid cell types.

Together, 2xHKBCG immunization did not protect against BCG infection and did not induce an effective immune response; rather, it tended to enhance lung bacterial growth. In contrast, an equivalent dose of 2xHKMtb immunization led to lower bacterial burden in the lungs after BCG infection and resulted in both M-MDSC and myeloid effector cells. These results suggest that 2xHKMtb vaccination may induce robust immunity against BCG despite the parallel M-MDSC induction.

### Expansion and activation of DC subsets in the lungs of Mtb-immunized mice

**Figure 3:**
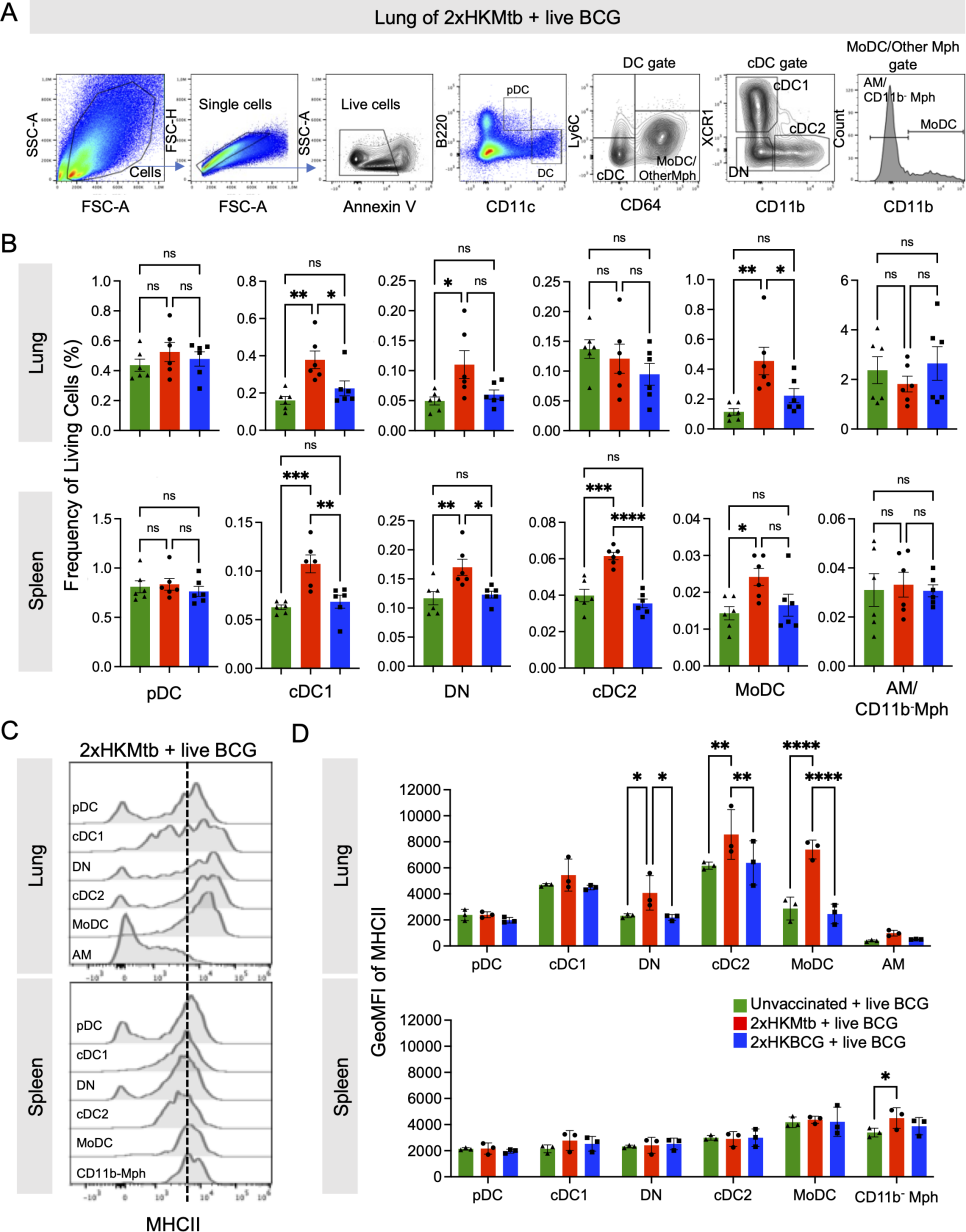
Live BCG-infected lungs of 2xHKMtb-immunized mice show high activation of several dendritic cell and monocyte subsets. **(A)** Gating strategy to identify DC subsets. Plasmacytoid DC (pDC) were defined as B220^+^ CD11c^low^. CD11c^hi^ DC were divided into Ly6C^-^ CD64^+^ Monocyte-derived DC (MoDC), other Mph and Ly6C^-^ CD64^-^ conventional DC (cDC). The Monocyte-derived DC (MoDC) and other Mph gate was further subdivided into CD11b^+^ MoDC, and CD11b^-^ AM (in lung) or CD11b^-^ Mph (in spleen). The cDC gate was further subdivided into XCR1^+^ CD11b^-^ cDC1 and XCR1^-^ CD11b^+^ cDC2. Cells negative for XCR1 and CD11b were gated as double negative DC (DN). **(B)** Frequencies of living cells in specified gates of lung and spleen. Gates as in **(A, C)**. Histogram overlays showing MHCII expression of indicated cell subsets in lung and spleen. **(D)** GeoMFI of MHC II in indicated gates of lung and spleen. Gates as in **(A, B)**: Statistics by ordinary one-way ANOVA with multiple comparisons and Tukey's post test. n=12 biological replicates pooled 2 by 2 (therefore n=6), 2 independent experiments. **(D)** Statistics by ordinary two-way ANOVA with multiple comparisons and Tukey's post test. n=6 biological replicates pooled 2 by 2 (therefore n=3). Not significant (ns); *p<0.05; **p<0.01; ***p<0.001; ****p<0.0001. Dotted lines separate MHC II^low^ from MHC II^high^ expression for immature and mature DC subsets, respectively.

To corroborate the hypothesis that live BCG infection after 2xHKMtb vaccination triggers a switch towards immunogenic myeloid cell phenotypes, we investigated the responses of myeloid cells, including DC subsets, after 2xHKMtb or 2xHKBCG immunizations and live BCG infection. The gating strategy was adopted from our previous work (41) and allowed us to separately analyze plasmacytoid DC (pDC), MoDC, conventional DC1 (cDC1), conventional DC2 (cDC2), and XCR1/CD11b-double negative (DN) DC, and different Mph subsets (CD11c^high^ CD11b^-^ in the spleen and CD11c^high^ CD11b^-^ AM in the lungs) (**Figure 3A**). 2xHKMtb immunization followed by BCG infection resulted in enhanced frequencies of cDC1, MoDC, and DN populations in the spleen and lungs, in addition to cDC2 in the spleen. In contrast, 2xHKBCG immunization followed by BCG infection showed no effect (**Figure 3B**).

MHC II expression of the DC was further used as an activation marker (**Figures 3C, D**). In the lungs, 2xHKMtb immunization induced the highest MHC II expression in MoDC, cDC2, and DN populations (**Figures 3C, D**) and moderate levels in cDC1. In contrast, no MHC II expression was observed in the spleen, except moderately in CD11b^-^ Mph under 2xHKMtb immunization conditions (**Figures 3C, D**). Again, no DC activation was observed when mice were immunized with 2xHKBCG and infected with live BCG in the lungs and spleen (**Figures 3C, D**). These data indicate that DC subsets are activated in BCG-infected animals previously vaccinated with 2xHKMtb, but not with 2xHKBCG, suggesting a greater potential of 2xHKMtb vaccination to elicit protective T cell responses.

### Mtb immunization before BCG infection results in enhanced lung CD4^+^ and CD8^+^ T cell responses

**Figure 4:**
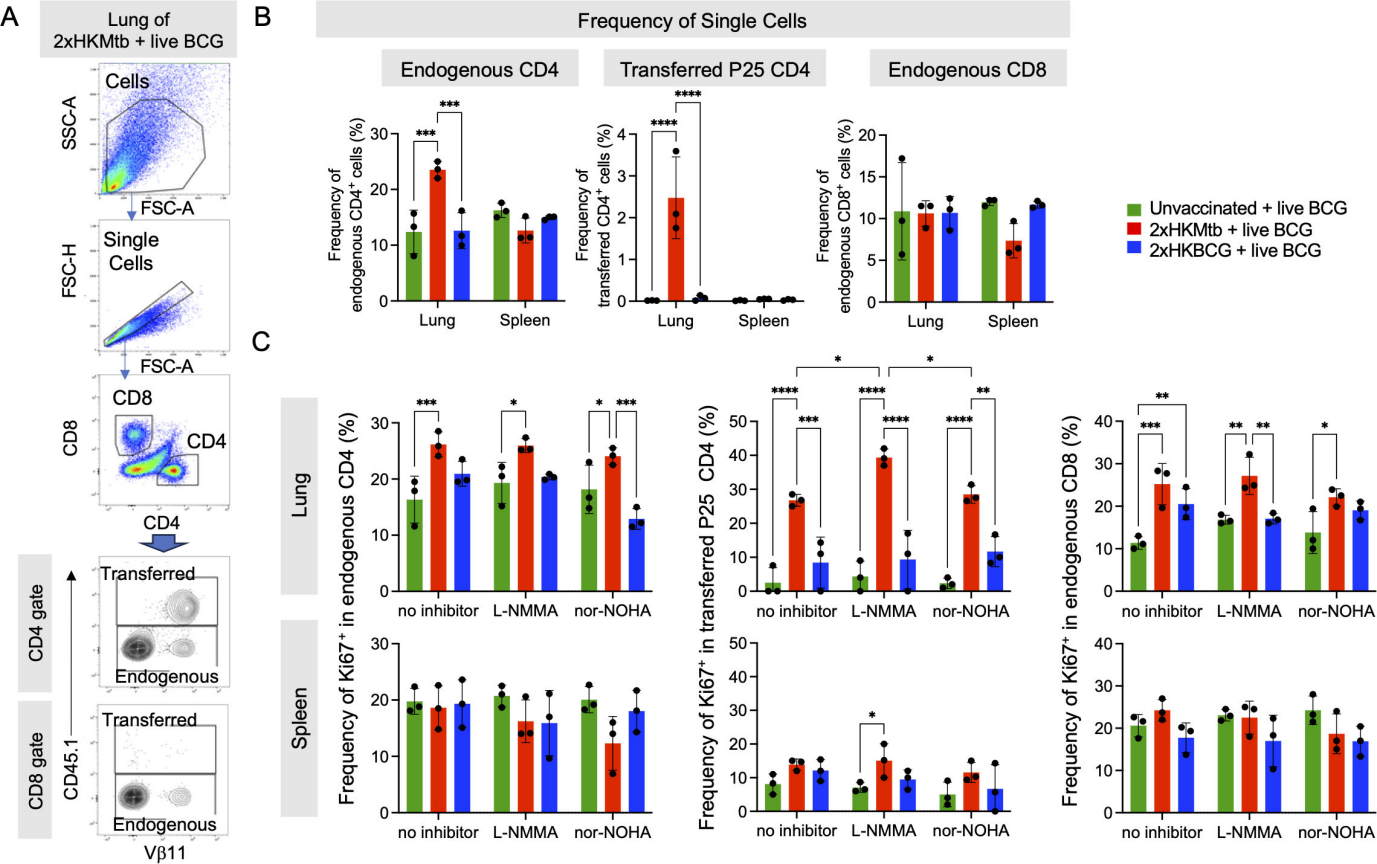
Enhanced CD4+ and CD8+ T cell immune response in the lungs of 2xHKMtb-immunized and live BCG-infected mice. **(A)** Representative lung flow cytometry plots and gating strategy to identify endogenous and transferred CD4 and CD8 T cells. Initial gating included FSC-A/SSC-A and doublet exclusion. CD4^+^ and CD8^+^ lymphocytes were segregated based on their surface expression of CD4 or CD8, respectively. Transferred CD4^+^ transgenic P25 T cells were gated as CD45.1^+^ based on their expression of the congenic marker. Endogenous CD4^+^ and CD8^+^ T cells were gated as negative for CD45.1. **(B)** Frequencies of single cells in lung and spleen endogenous CD4, transferred P25 CD4 and endogenous CD8 gates. Gates as in **(A, C).** Bulk lung and spleen cells were stimulated at day 47 with P25 peptide and cultured for 3 days with or without iNOS inhibitor L-NMMA or Arg1 inhibitor nor-NOHA. T cell proliferation was measured by flow cytometry and is depicted as frequency of Ki67^+^ within endogenous CD4, transferred CD4 and endogenous CD8 T cell subsets. Statistics with two-way ANOVA with multiple comparisons and Tukey's post test. n=6 biological replicates pooled 2 by 2 (therefore n=3). *p<0.05, **p<0.01, ***p<0.001, ****p<0.0001.

It remained to be determined how the mixed induction of immunostimulatory and suppressive myeloid cells would translate into adaptive immunity. To test this, antigen-specific versus polyclonal endogenous T cell responses were investigated in the 2xHKMtb- or 2xHKBCG-vaccinated and BCG-infected mice. Mice received CD45.1^+^ splenocytes from P25 mice carrying a TCR-transgene in CD4^+^ T cells with specificity for peptide 25 of mycobacterial Antigen 85B (42). This enabled us to follow the peptide-specific T cell expansion against Mtb and BCG and compare it to the endogenous T cell repertoire (**Figure 4A**). A massive expansion of antigen-specific P25 and endogenous CD4^+^ T cells was observed in the lungs of Mtb-immunized but not BCG-immunized mice infected with BCG (**Figure 4B**). CD8^+^ T cell populations showed no change in immunized mice compared to unvaccinated controls. To test the P25 and endogenous T cell proliferation, bulk lung and spleen cells were re-stimulated *in vitro* with peptide P25 for 72h, followed by assessment of the Ki67^+^ proliferating cells. P25 T cells proliferated heavily only in the 2xHKMtb vaccinated animals. In contrast, T cell responses of animals that received 2xHKBCG were not elevated and remained at a very low level, similar to those of unvaccinated mice (**Figure 4C**). Significantly increased proliferation of CD8^+^ and CD4^+^ endogenous T cells could also be detected in the lungs, but not spleen, in mice after 2xHKMtb immunization (**Figure 4C**). Interestingly, P25 T cell proliferation in the lungs was further enhanced by adding an iNOS inhibitor N^G^-Methyl-L-arginine acetate salt (L-NMMA) but not an Arg1 inhibitor Nω-hydroxy-nor-arginine (nor-NOHA) to the culture, suggesting the presence of iNOS-mediated suppressive activity. 2xHKBCG immunization followed by BCG infection only increased CD8^+^ T cell proliferation upon such *in vitro* re-stimulation (**Figure 4C**).

These findings indicate mycobacteria-specific P25 and endogenous CD4^+^ T cell expansion in the lungs as the major infected organ after 2xHKMtb immunization and BCG infection. The increased proliferation of lung-derived T cells following iNOS inhibition corresponds with the reduced frequency and activation of M-MDSC observed in these mice.

### Depletion or inhibition of M-MDSC before BCG infection further lowers bacterial load in the lungs and enhances cDC1 frequencies and T cell responses

**Figure 5:**
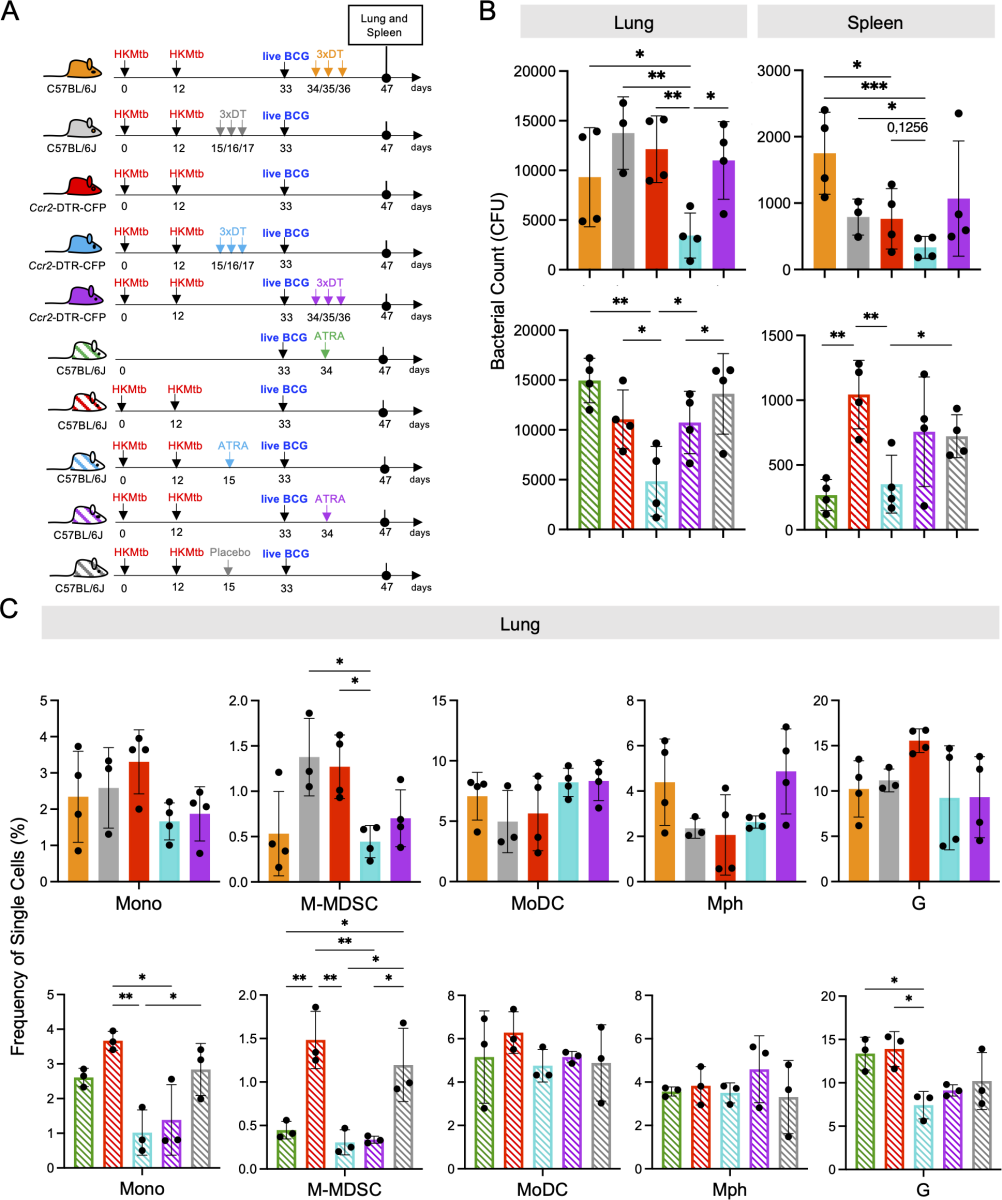
CCR2^+^ cell depletion or All-trans retinoic acid (ATRA) treatment prior to intranasal live BCG-infection further decreases lung bacterial burdens and M-MDSC infiltration in 2xHKMtb-immunized and live BCG-infected mice. **(A)** Schemes showing the genetic and pharmacological interference with M-MDSC. Mice remained unvaccinated or were immunized s.c. with 10^6^ CFU heat-killed Mtb/IFA at day 0 followed by a booster dose at day 12. On day 33, all mice were infected i.n. with 10^7^ CFU live BCG. Ccr2-DTR-CFP mice were treated with DT before or after live BCG infection. Similarly, C57BL/6 mice were treated with ATRA or placebo pellets before or after live BCG infection. Lungs and spleens were harvested and analyzed at day 47. **(B)** Bacterial burdens in whole lungs and spleens. **(C)** Frequencies of single cells in specified gates of lung and spleen. Gates as in **Supplementary Figure 1A**. **(B)** Statistics by unpaired, two-tailed t-test. n=4 biological replicates, 2 independent experiments. **(C)** Statistics by ordinary one-way ANOVA with multiple comparisons and Tukey's post test. n=3-4, 2 independent experiments. *p<0.05; **p<0.01; ***p<0.001.

Re-stimulation of mycobacteria-specific P25 T cells from the lung indicated that M-MDSC were still partially suppressing the T cell response *in vitro*. To demonstrate that M-MDSC are also active *in vivo*, we used *Ccr2*-DTR-CFP mice, where all CCR2^+^ monocytes and monocyte-derived cells can be depleted by injection of Diphtheria toxin (DT). We vaccinated with 2xHKMtb and performed the depletion after M-MDSC induction, but before the BCG infection, or after the BCG infection (**Figure 5A**). Depleting CCR2^+^ cells after M-MDSC induction but before the BCG infection (days 15, 16, 17) further reduced the bacterial loads in the lungs and spleen, although this did not reach statistical significance in the spleen (**Figure 5B**). In contrast, depletion of CCR2^+^ cells after BCG infection at the peak of cDC activation and subsequent T cell response (days 34,35,36) did not further reduce the bacterial load in the lungs or spleen, similar to DT injections as a control into wild-type (WT) mice (**Figure 5B**).

**Figure 6:**
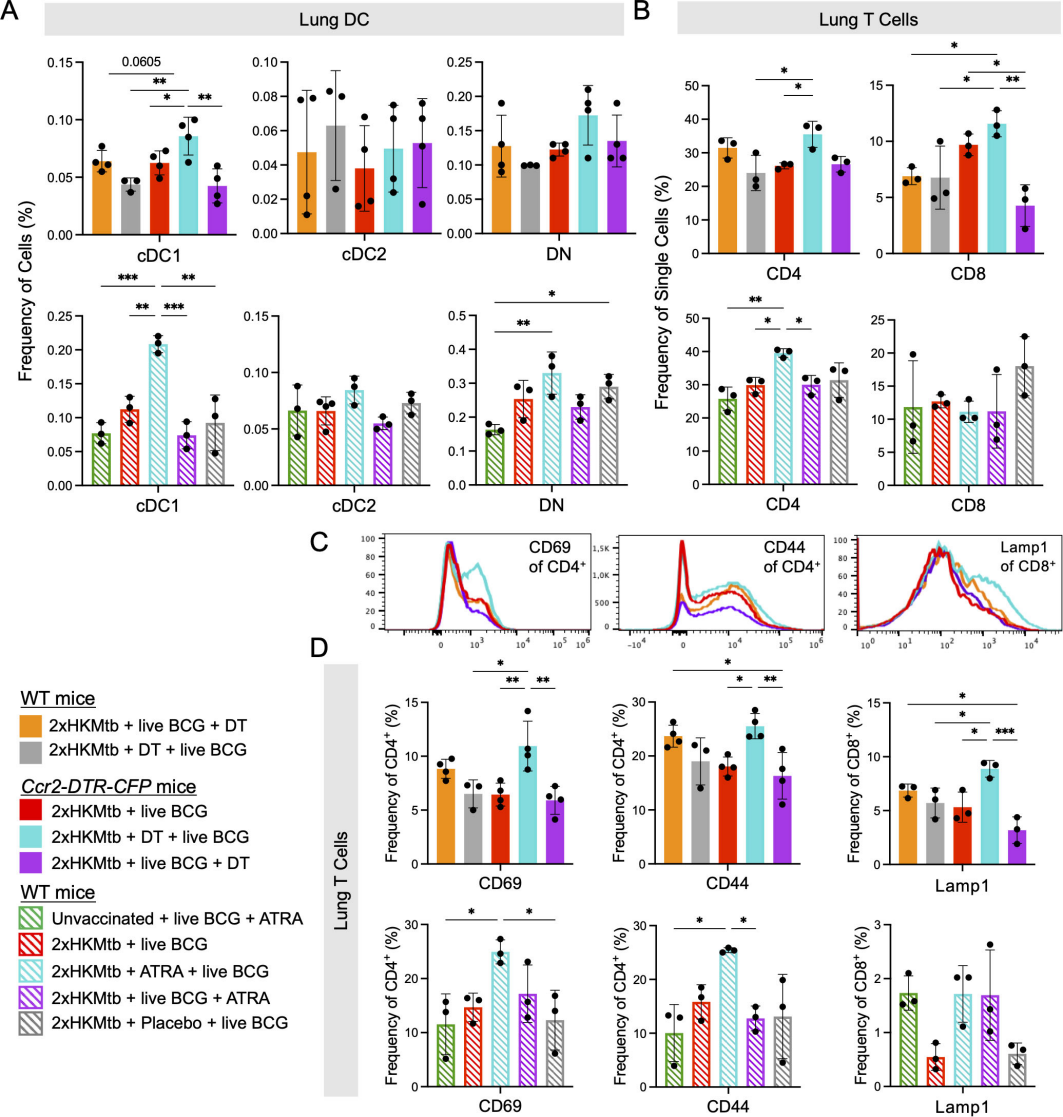
CCR2^+^ cell depletion or ATRA treatment prior to intranasal live BCG infection increases frequency of cDC1 and enhances activation of T cells in the lung. *Ccr2*-DTR-CFP or WT mice were immunized as indicated, infected and treated according the scheme shown in **Figure 5A**. **(A)** Frequencies of cells in specified DC populations in lung. Gates as in **Figure 4A**. **(B)** Frequencies of single cells in the CD4 and CD8 T cell gates. Gates as in **Figure 5A**. **(C)** Representative histograms showing expression of CD69 and CD44 activation markers in the CD4^+^ T cell gate and of Lamp1 in the CD8^+^ T cell gate from indicated conditions. **(D)** Frequencies of CD69^+^ and CD44^+^ cells in CD4^+^ T cell gate and Lamp1^+^ cells in the CD8^+^ T cell gate. Statistics by ordinary one-way ANOVA with multiple comparisons and Tukey's post test. n=3-4, 2 independent experiments. *p<0.05; **p<0.01; ***p<0.001.

To confirm the beneficial effect of genetic M-MDSC depletion, we next employed the pharmacological MDSC inhibitor ATRA (43) before and after BCG infection (**Figure 5A**). Similar to CCR2^+^ cell depletion, ATRA treatment led to a significant reduction in the bacterial loads in the lungs and spleen (**Figure 5B**). Since the major effects of DT and ATRA on the bacterial load were observed in the lungs, we tested whether this correlated with the successful depletion of M-MDSC and how other myeloid cells were affected. As expected, M-MDSC declined after genetic or pharmacological interference with MDSC, along with monocytes, but not MoDC, Mph, or granulocytes (**Figure 5C**). Then, we investigated whether genetic and pharmacological interference with M-MDSC induction promotes cDC and T cell responses. The results indicate that cDC1 selectively increased at the early interference time points (**Figure 6A**). This correlated with elevated CD4^+^ T cell frequencies (**Figure 6B**) and higher expression of the activation markers CD69 and CD44 on the CD4^+^ T cell subset, while CD8^+^ T cells showed higher expression of the cytotoxicity-related marker Lamp1 (**Figures 6C, D**). Moreover, serum analysis of mice treated with ATRA showed elevated concentrations of inflammatory cytokines (**Supplementary Figure 4**). These data align with the results above, showing that MDSC depletion increased myeloid cell and T cell frequencies, activation, and function.

Together, the depletion experiments support the concept that interfering with M-MDSC shortly after their induction by 2xHKMtb vaccination further lowers lung bacterial load by promoting cDC1, resulting in better expansion and activation of CD4^+^ and CD8^+^ T cells.

### Lung infiltrates decline after ATRA treatment and redirect T cells towards bacteria

**Figure 7:**
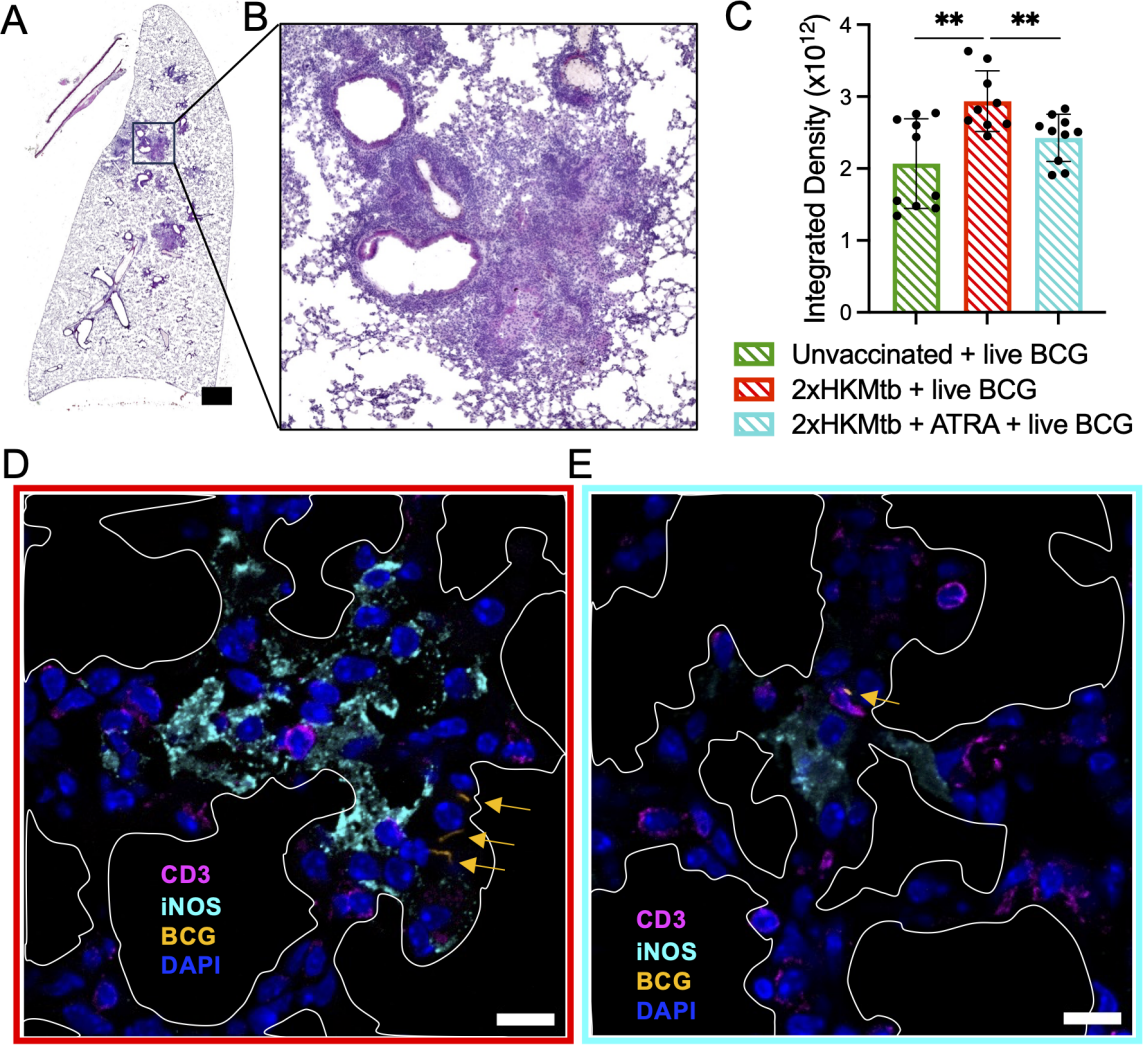
ATRA treatment lowers cell infiltration in the lungs, reduces iNOS^+^ areas, thereby promoting CD3^+^ T cell contacts to BCG bacteria. The indicated selected groups of WT mice were immunized and infected according to the scheme shown in **Figure 5A**. **(A)** Representative image of total lung section stained with H&E from a mouse immunized with 2xHKMtb, treated with ATRA, and infected with live BCG. Scale bar represents 1500µm. **(B)** Zoomed image of **(A)** that shows densely packed cell nuclei illustrating areas of massive cell infiltration. **(C)** Quantification of lung cell infiltrates of indicated groups as integrated density based on H&E staining. **(D, E)** Lungs from the indicated groups were collected at day 47 and cryosections were stained for CD3, iNOS, and DAPI to indicate T lymphocytes, M-MDSC and cell nuclei, respectively. White marked areas indicate alveolar spaces. Yellow arrows indicate dsRed-BCG fluorescence. Scale bars represent 10µm. Statistics by unpaired, two-tailed t-test. n=2 biological replicates, 5 sections per biological replicate each corresponding to a different depth of the tissue. **p<0.001.

To better understand the histological changes associated with M-MDSC depletion, we analyzed lung sections from WT mice vaccinated with 2xHKMtb, and treated or not with ATRA, prior to live BCG infection. These sections were compared with those from unvaccinated but BCG-infected mice. Analyses of hematoxylin and eosin (H&E)-stained sections indicated larger areas of infiltration in the mice vaccinated with 2xHKMtb compared to those that were only infected. ATRA treatment reduced the infiltrates to levels seen in mice that were solely BCG-infected (**Figures 7A–C**, **Supplementary Figure 5**). More detailed analysis using confocal fluorescence microscopy revealed larger areas of iNOS staining without ATRA treatment. Some bacteria were found in association with iNOS^+^ cells, suggesting bacterial killing (**Supplementary Figures 6A, C**). However, most iNOS^+^ cells were not in contact with bacteria but in conjunction with or in proximity to CD3^+^ T cells (**Figure 7D**). ATRA treatment resulted in fewer and smaller iNOS^+^ areas, and CD3^+^ T cells were now located closer to the infiltrating BCG bacteria (**Figure 7E**, **Supplementary Figures 6B, D**). Together, these data support our findings from above, suggesting that the elevated iNOS production in the lungs is associated with M-MDSC-mediated suppression of T cells rather than killing of bacteria. ATRA treatment reverses this situation and improves T cell responses against the bacteria.

## Discussion

Our previous data indicated that immunizing mice twice with heat-killed Mtb in IFA massively induced M-MDSC in the spleen, which entered the white pulp upon a microbial challenge to kill DC, thereby interfering with the induction of an immune response (15). Here, we tested additional bacterial strains using the same immunization strategy (prime-boost with heat-killed bacteria), and found that qualitative differences exist between bacterial species in expanding M-MDSC. The question remained whether specific molecules expressed by some bacteria, such as Mtb, are responsible for M-MDSC expansion. There are major genetic differences between Mtb and BCG, most prominently known as "Regions of Difference" (RD) (44). The RD1 region, present in virulent Mtb and Mbov but absent in avirulent BCG (45), has received the most attention, especially because it encodes the secreted molecules ESAT-6 and CFP-10, which represent major virulence factors and important antigens for T and B cells (5). However, no role for RD1 in MDSC induction has been shown, and we did not see differences between BCG and Mbov in their capacity to induce M-MDSC. Apart from the most widely studied RD1 region, the additional Mtb gene regions RD7, RD8, RD9, and RD10 are not present in BCG or Mbov (46), which may encode for Mtb-specific M-MDSC-inducing molecules. PhoP deletion is one of two major modifications of the highly promising MTBVAC vaccine candidate that is based on the Mtb H37Rv strain (47, 48). Its high immunogenicity may therefore be due to a lack of M-MDSC induction by the PopP deletion. However, the Mtb H37Ra strain used here is devoid of the PhoP-encoded sulfolipids and diacyl/polyacyl trehalose. Thus, this molecule group cannot account for the Mtb-specific M-MDSC induction observed here. The MTB64 protein of Mtb, also present in Mbov but not BCG, has been proposed to induce murine MDSC-like cells *in vitro* (49), which awaits confirmation *in vivo*. Thus, whether a specific Mtb molecule can induce MDSC is still open. However, the fact that several different bacterial vaccines could induce MDSC, as shown here, argues for a more general common mechanism.

Induction of MDSC may be driven by host factors such as inflammation or damage-associated molecular patterns to protect tissue integrity. We showed before that not only exogenous products from pathogens but also a cytokine cocktail of IL-1β, TNF, IFN-γ, and IL-10, representative for an inflammatory response, could activate M-MDSC *in vitro* to the same extent as LPS/IFN-γ (50). Repetitive injections of GM-CSF into mice induced similar splenomegaly and M-MDSC induction as seen here after 2xHKMtb injection (50). Since pathogens or their products also induce an inflammatory response *in vivo*, the pathogen-induced M-MDSC induction may represent an indirect effect mediated by endogenous cytokines. This would align with the concept that MDSC induction limits overshooting immunity in response to high levels of cytokines, e.g. by activated T cells (51). In support of this, our data indicate that depletion of M-MDSC by ATRA results in the up-regulation of cytokines in the serum. Histological analysis of the lung showed increased cellular infiltrates after 2xHKMtb vaccination without ATRA. T cells appeared in close proximity to or within large areas of iNOS^+^ cells. The fact that many iNOS^+^ cells were not directly infected by BCG but in adjacent areas may suggest that iNOS induction could be mediated by endogenous pro-inflammatory cytokines in the infected areas, rather than or in addition to direct contact with the bacteria.

2xHKMtb immunizations led to massive M-MDSC induction in both the lungs and spleen. However, after BCG infection in these mice, we observed a decreased bacterial load in the lungs but an increase in the spleen. The lower bacterial load in the lungs correlated with higher frequencies and activation levels of several DC subsets and T cells compared with the spleen. Thus, the local lung infection attracted both pro- and anti-inflammatory cells. The suppressor activity of M-MDSC isolated from these lungs indicated a parallel competing stimulation and inhibition of T cell responses. In this case, the dominant stimulatory response led to a lower bacterial load. Such competition between infiltrating pro- and anti-inflammatory cell types is well documented in tumors where infiltrating DC and T cells are controlled by MDSC, frequently tipping the balance towards increased tumor growth (9, 52). Similarly, high frequencies of M-MDSC were already present in the spleen when BCG was disseminated to this organ, and thereby suppression dominated.

Surprisingly, the 2xHKBCG vaccination failed to induce signs of splenomegaly or an innate or adaptive immune response. Lungs and spleens even showed a trend towards higher bacterial loads than the controls. In the 2xHKBCG immunization, the same dose of BCG was used as Mtb to compare with the established 2xHKMtb immunization for M-MDSC induction. It has been previously observed in humans that there is no significant change in control of BCG outgrowth after revaccination with BCG (53). In addition, the live BCG vaccine has been shown to be more immunogenic than the vaccine with killed BCG vaccines (54, 55). This may be due to its longer persistence in the host, the possibility of dissemination, and increased tissue damage, rarely observed during immunodeficiency states (56, 57).

Although BCG vaccination is widely used against TB, no vaccine based directly on Mtb has yet been approved. However, MTBVAC, an Mtb-based candidate, has now advanced to Phase III clinical trials (58). Immune evasion mechanisms in Mtb have been discussed as the main reason for this failure (59, 60). To improve the vaccination success against TB, adjunct host-directed therapies have been proposed (21, 61). Here, we tested whether the protection against BCG infection achieved by 2xHKMtb immunization could be further improved by interfering with M-MDSC. While previous studies demonstrated that ATRA can treat Mtb-infected mice successfully (8, 23, 24), we show here that it can also be used as an adjunct to an Mtb-based vaccine. At early time points (d15/16/17), when M-MDSC induction peaked, both a genetic approach targeting CCR2^+^ cells and a pharmacological approach using ATRA reduced the frequency and suppressive function of M-MDSCs. Notably, the effect of ATRA has been previously reported in tumor-bearing mice, where it produced similar outcomes (22). Here, ATRA treatment also elevated levels of inflammatory cytokines and was associated with higher frequencies of cDC1 and CD4^+^ T cells in the lungs. Intervention at later time points (d34/35/36) following live BCG infection failed to reduce bacterial counts, enhance DC numbers, or improve T cell responses despite reducing the M-MDSC frequencies in the lungs. While DT depletion of CCR2^+^ cells and their descendants affects both pro-inflammatory MoDC and suppressive M-MDSC, ATRA is believed to affect only MDSC. Since the genetic and pharmacological approaches behaved very similarly here, this, however, suggests that ATRA does not exclusively act on suppressive cells but also inhibits the differentiation of pro-inflammatory cells such as DCs. This seems to contrast with what has been observed in human studies, where DC vaccination against small cell lung cancer was successfully combined with ATRA treatment (28). However, the vaccines in this study were already mature DC, which did not require differentiation from progenitors or monocytes. ERK1/2 signaling, a major pathway responsible for ATRA-mediated inhibition of MDSC differentiation (22), plays an important role in DC survival but not maturation (62). Thus, the optimal time point to interfere with M-MDSC in a vaccine setting appears to be in conjunction with the vaccine but not after infection, where pro-inflammatory DCs are generated from progenitors or monocytes. Recent clinical studies to interfere with MDSC by ATRA as an adjunct treatment with checkpoint inhibitors in tumor patients have shown encouraging results (27, 63).

The current BCG vaccine is often administered immediately after birth. In both mice and humans, high levels of G-MDSC and M-MDSC have been observed in newborns, but these cells disappear within two weeks (64). These MDSC play a key role in managing necrotizing enterocolitis during the initial microbiological colonization of the intestine and subsequent immune system development (64). Since the absence of MDSC enhances vaccine effectiveness, as shown here, it might be advisable to delay TB vaccination until after MDSC levels decline around week 3 post-birth. Although ATRA is used to treat pediatric leukemia (65), its use during childhood may not be advisable alongside a TB vaccine due to potential severe side effects, like the life-threatening 'differentiation syndrome' (66). For adult TB vaccine recipients, the ATRA combination may offer clear benefits, since their immune response against the vaccine and memory cell development will benefit from MDSC depletion.

**Figure 8:**
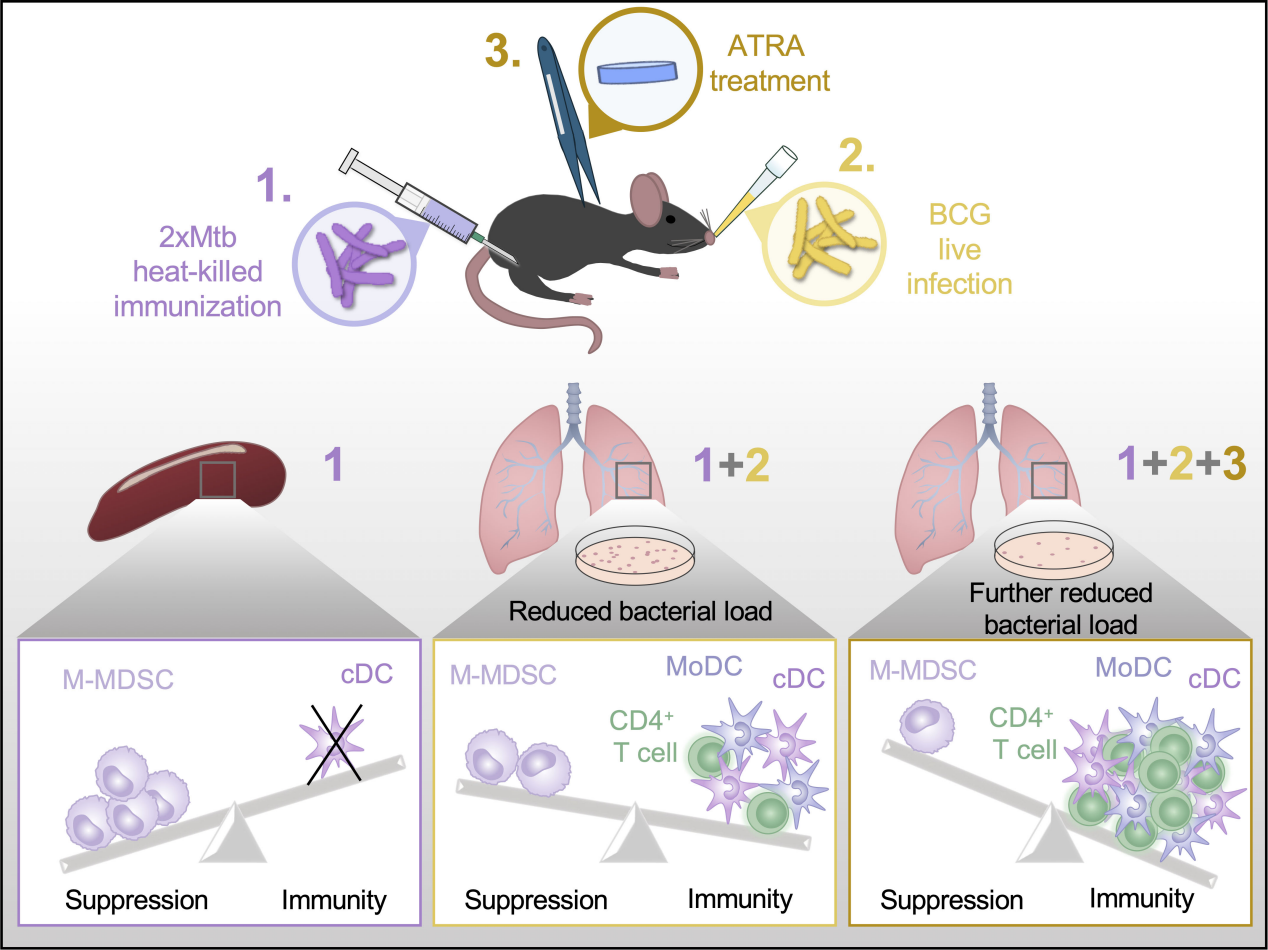
Graphical abstract. A summary of our main finding that ATRA treatment can improve Mtb vaccine success is depicted schematically.

In conclusion, our work provides several new insights into vaccination with mycobacteria. First, 2xHKMtb immunization showed the highest potential to induce M-MDSC out of the bacteria tested. Second, despite the induction of M-MDSC after 2xHKMtb vaccination, it also induced a substantial immune response that lowered lung bacterial loads in our setting. Third, ATRA treatment during 2xHKMtb vaccination to interfere with M-MDSC further improved the immune response and increased protection against infection (**Figure 8**). Together, these results may encourage the future use of ATRA in human vaccination studies against TB and other pathogens where MDSC induction has been observed (67).

## Materials and methods

### Mice and interference with M-MDSC

P25 transgenic mice (42) were kindly provided by Ulrich Schaible, Borstel, Germany. P25 mice were crossed with B6.CD45.1 congenic mice to obtain B6.P25.CD45.1 mice. C57BL/6J WT mice were initially purchased from Charles River, Sulzfeld, Germany. All mice were bred in our animal facilities and kept under specific pathogen-free conditions. Both sexes were used for experiments at an age of 6–12 weeks. Georg Gasteiger kindly provided *Ccr2*-DTR-CFP mice. DT (Unnicked, *Corynebacterium diphtheriae*, Merck, Darmstadt, Germany) was injected i.p. at indicated time points at a dose of 0.5µg per injection in PBS. Pellets containing ATRA or equivalent placebo were applied s.c. (5mg/p, 21-day release, Innovative Research of America, Sarasota, FL) at the indicated time points. Before BCG infection and ATRA implantation, all mice were anesthetized by i.p. injection of Ketamine (100µg/g) and Medetomidine (1µg/g) mixture in 0.9% NaCl solution. Finally, as a reversal of anesthesia, mice were inoculated i.p. with 12.5µg/g Anti-sedan (active ingredient Atipamezole Hydrochloride) in 0.9% NaCl solution. The euthanization of animals was performed in a CO_2_ chamber. All animal euthanizations, treatments, and experiments were performed according to German animal protection law and after approval and under the control of the local authorities (Regierung von Unterfranken, AZ 55.2-2531.01-64/11 and 55.2.2-2532-2-1408).

### Bacterial immunization

All vaccines were applied as heat-killed bacteria at a dose of 10^6^ CFU, which were mixed in IFA (purchased from MilliporeSigma) or alum (purchased from Brenntag) and emulsified in an equal amount of PBS. A creamy preparation was achieved by passing the mixture continuously through 2 syringes connected by a medical valve 3-way stopcock for 10 minutes. C57BL/6J mice were immunized s.c. by injecting 200µl bacteria/IFA-PBS or, where indicated, bacteria/alum-PBS emulsion into one flank. Booster immunization was administered s.c. by introducing 200μl emulsion into the other flank. When indicated, 10^7^ bulk spleen cells from B6.P25.CD45.1 mice in a total volume of 50µl per mouse were injected i.v. into mice tail veins.

### Bacteria and infection

Heat-killed and lyophilized Mtb was purchased from Difco. Live BCG was kindly provided by Nathalie Winter, Pasteur Institute Paris, France; was of strain Pasteur, expressing red fluorescence (dsRed) (68). Live Msm was kindly provided by Sebastian Geibel, Würzburg, Germany. Heat-killed List was provided by Thomas Hünig, Würzburg, Germany. Heat-killed MtbM and Mbov were kindly provided by our Microbiology Department. Both Mtb preparations (Mtb and MtbM) used in this study were of the H37Ra strain. BCG and Msm were cultured in Middlebrook 7H9 broth medium (MilliporeSigma) supplemented with 0.05% Tween-80 (MilliporeSigma), 0.05% glycerol (Roth), 10% ADC (Albumin, Dextrose, Catalase, SERVA), and 30µg/ml Hygromycin (MilliporeSigma) at 35°C until reaching the exponential growth phase. The bacterial suspension was then centrifuged at 30 G for 10 minutes, quantified, used, or stored in 20% glycerol at -20°C. Heat-killing was performed by placing aliquots in a water bath at 80°C for 60 minutes. All infections were performed using live BCG. Anesthetized mice were inoculated i.n. with 20µl droplets containing 5 x 10^8^ CFU/ml.

### Evaluation of infection by determination of bacterial load

Lung and spleen bacterial loads of BCG-infected mice were determined by plating serial dilutions of whole-organ homogenates on Middlebrook 7H11 agar medium (MilliporeSigma) enriched with ADC. Petri dishes were covered with permeable parafilm and incubated at 37°C in humidified air containing 5% CO_2_. Bacterial colonies were counted 2–3 weeks post-incubation.

### Flow cytometry

The murine directly conjugated antibodies CD11b-Alexa Fluor 700/-Brilliant Violet 650 (M1/70), Ly6G-Brilliant Violet 650/-APC-Cy7 (1A8), Ly6C-Brilliant Violet 510/-Brilliant Violet 785 (HK1.4), CD11c-PE-Cy7 (N418), CD16-2-Brilliant Violet 421 (9E9), B220-Pacific Blue (RA3-6B2), XCR1-APC (ZET), CD64-PE (X54-5/7.1), MHC II-Alexa Fluor 700 (M5/114.15.2), CD4-PerCPCy5.5/-Pacific Blue (GK1.5), CD8-Alexa Fluor 488/-Brilliant Violet 510 (53-6.7), Vβ11-APC (RR3-15), CD45.1-Pacific Blue (A20), Ki67-PE (11F6), CD69-PE-Cy7 (H1.2F3), CD44-Alexa Fluor 488 (IM7), LAMP1/CD107a-PE (1D4B), PD-L1/CD274-PE (10F.9G2), Annexin V-FITC were all purchased from Biolegend. S100A9-APC (2B10) was bought from BD Pharmingen. iNOS-Alexa Fluor 488 (CXNFT), Arg1-APC (A1exF5), Fixable Viability Dye-eFluor 780 (<ns/>65-0865) were obtained from eBioscience. Surface staining was performed by incubating 10^6^ cells in 50µl FACS buffer (0.1% BSA in PBS) containing fluorescent-conjugated antibodies for 30 minutes at 4°C. To block unspecific antibody binding to surface FcγRII/III, 50% supernatant from the 2.4G2 hybridoma was used in FACS buffer. Cells were then fixed with 2% fresh formaldehyde (Roth) for 20 minutes at room temperature. When an intranuclear staining had to follow, cells were instead fixed by Fix/Perm buffer, diluted 1:3 with Fix/Perm diluent (eBioscience). For intracellular detection, cells were permeabilized and stained in 50µl Perm buffer (eBioscience) with fluorescent-labeled antibodies for 1 hour at room temperature. Samples were washed with Perm buffer, resuspended in FACS buffer, and measured with the Attune NxT (Thermo Fisher Scientific). Data were analyzed by FlowJo 10 (Tree Star).

### Activation of M-MDSC

To activate M-MDSC *ex vivo*, spleen and lung cell suspensions were seeded in 24-well plates at a density of 1 x 10^6^ cells/ml per well. They were cultured in the presence of 100ng/ml Lipopolysaccharide (LPS, MilliporeSigma) and 100U/ml IFN-γ (Immunotools) for 16 hours as described (12, 50).

### Suppressor assay

Bulk spleen and lung single-cell suspensions were seeded in 96-well plates at a concentration of 2 x 10^5^ cells/well in 200µl RPMI medium containing 10% FCS and stimulated with soluble anti-CD3/anti-CD28 antibodies at a final concentration of 2.5µg/ml each. In P25 spleen cell transfer experiments, cells were stimulated with 10µg/ml P25 peptide (FQDAYNAAGGHNAVF) instead. At day 3, proliferation was measured by flow cytometry via Ki67 detection separately in CD4^+^ and CD8^+^ T cell subsets. L-NMMA (500 μM, MilliporeSigma) and nor-NOHA (50μg/ml, Cayman Chemicals) were added to the cultures when required.

### Legendplex analysis

On the day of sacrifice, blood was collected by cardiac puncture, and serum was separated by centrifugation at 3.6G for 30 minutes at 4°C. The samples were stored at -20°C until thawed and tested for the presence of cytokines using the LEGENDplex™ Mouse Inflammation Panel 13-plex kit (Biolegend) following the manufacturer's protocol. The samples were then measured with the Attune NxT (Thermo Fisher Scientific) and analyzed using the LEGENDplex™ online software (Biolegend).

### Preparation of frozen lung sections

The thorax of the euthanized mice was opened, and the left lung lobe was removed. The bronchus was sealed, and the trachea was cannulated with a 22-G venous catheter. A 1:1 mixture of 10% (w/v) sucrose in PBS and O.C.T.™ Tissue-Tek^®^ (Sakura Finetek Europe) was gently injected into the right lung lobe to inflate it fully. After inflation, the trachea was ligated, and the right lung lobe was carefully dissected out along with the heart. The lung lobe was then placed in a Tissue-Tek^®^ Cryomold^®^, fully submerged in O.C.T.™ Tissue-Tek^®^, and rapidly frozen on dry ice.

Frozen lung samples were sectioned at 7 µm for immunofluorescent staining and at 10 µm for H&E staining using Kawamoto adhesive film (69). Sections were mounted on Epredia™ SuperFrost Plus™ Adhesion glass slides (Thermo Fisher Scientific) for subsequent analysis.

### Immunofluorescence staining

For immunofluorescence staining, sections were thawed and rehydrated in PBS, then fixed in 4% fresh paraformaldehyde (PFA) in PBS. Permeabilization was performed using 0.5% Triton X-100. Sections were then blocked with 5% goat serum, 0.1% Tween-20, and an avidin/biotin blocking kit (BioLegend). Primary antibodies were applied as CD3-biotin (BioLegend, 145-2C11) and iNOS-Alexa Fluor 488 (Invitrogen, FJK 16s). Corresponding biotinylated targets were detected using streptavidin-Alexa Fluor 647 (ThermoFisher). Finally, slides were mounted with DAPI-containing medium (Sigma-Aldrich) to visualize nuclei.

### Confocal laser scanning microscopy

Immunofluorescence images were taken using a confocal laser scanning microscope (LSM780, Carl Zeiss AG) with a 40x (NA 1.3) oil objective and ZEN software for image documentation. Image processing was performed with Fiji (ImageJ 2.16.0) software.

### H&E staining

For H&E staining, sections were thawed and rehydrated in 100% ethanol, then fixed in 4% PFA in PBS. After washing in distilled water, slides were immersed in Harris hematoxylin solution (Roth) for 1 minute and 30 seconds, followed by rinsing under running tap water. Sections were then stained with 0.5% eosin Y solution (Roth) for 40 seconds and again rinsed under running tap water. Dehydration was performed sequentially using 70%, 90%, and 100% ethanol, followed by clearing in xylene. Finally, slides were mounted using Eukitt^®^ mounting medium.

### Quantification of cell infiltration in lung tissue

For quantification of cell infiltrates, five sections were collected per lung lobe, with each section spaced 100 µm apart. Whole lung lobes were imaged using a Keyence BZ-X810 microscope equipped with either a 10x (NA 0.45) or 20x (NA 0.45) objective. Images were processed using Fiji software. Color deconvolution of H&E-stained sections was performed to separate the nuclear signal (purple channel), which was converted to an 8-bit grayscale image and then color-inverted. The lung lobe area was outlined (indicated in yellow), and integrated density was measured to quantify cell infiltration.

### Statistics

Figures were created and statistics were calculated using GraphPad Prism 10 software. Details on the statistical tests used are provided in the figure legends and include Student's t-test, one-way or two-way ANOVA as indicated. Before applying ANOVA, normal distribution was confirmed by D'Agostino and Pearson test or the Shapiro-Wilk test. P values of less than 0.05 were considered significant.

## Data Availability

The original contributions presented in the study are included in the article/**Supplementary Material**. Further inquiries can be directed to the corresponding author.
